# Extracellular vesicles of patients with acute-on-chronic liver failure induce mitochondrial dysfunction in T cells

**DOI:** 10.3389/fimmu.2025.1656692

**Published:** 2025-09-22

**Authors:** Mona-May Langer, Sabrina Guckenbiehl, Alina Bauschen, Helena Stadler, Farnusch Kaschani, Markus Kaiser, Bernd Walkenfort, Heiner Wedemeyer, Christian M. Lange

**Affiliations:** ^1^ Institute for Gastroenterology and Hepatology, University Hospital Essen, University Duisburg-Essen, Essen, Germany; ^2^ Department of Internal Medicine II, LMU University Hospital Munich, Munich, Germany; ^3^ Analytics Core Facility Essen, Center of Medical Biotechnology, Faculty of Biology, University Duisburg-Essen, Essen, Germany; ^4^ Chemical Biology, Center of Medical Biotechnology, Faculty of Biology, University of Duisburg-Essen, Essen, Germany; ^5^ Imaging Center Essen (IMCES), Electron Microscopy Unit (EMU), Medical Faculty of the University of Duisburg-Essen, Essen, Germany; ^6^ Department of Gastroenterology, Hepatology and Endocrinology, Hannover Medical School, Hannover, Germany

**Keywords:** systemic inflammation, exosomes, T cell exhaustion, organ failure, liver cirrhosis

## Abstract

**Background and Aims:**

Liver cirrhosis and in particular acute-on-chronic liver failure (ACLF) are characterized by systemic inflammation and dysfunctional immune responses. Extracellular vesicles (EVs) are important mediators of cell stress and inflammation, but their role in ACLF is unclear.

**Methods:**

Phenotype and immune function of EVs of patients with compensated liver cirrhosis, acute decompensation, or ACLF were characterized regarding particle size, concentration, surface markers, and RNA cargo. In addition, functional analyses were performed to assess the impact of EVs on T cells.

**Results:**

EVs of patients with liver cirrhosis showed lower expression of exosome-specific markers (e.g. CD9, CD63, CD81) than EVs of healthy individuals, carried a distinct cargo of proteins and small RNAs, and were in high frequency derived from liver cells based on their carriage of liver cell markers such as ASGPR1, CD248 or CD163. Of note, in ACLF the concentration of EVs decreased, and EVs in ACLF lost partially their differentiation and surface markers but were enriched in lncRNAs. In functional assays, EVs of patients with cirrhosis and ACLF induced changes in the composition of T cell populations like a loss of naïve and central memory T cells and an increase in effector memory T cells. Mechanistically, EVs decreased the viability of CD3^+^ T cells, which could be explained by an induction of mitochondrial dysfunction.

**Conclusion:**

Liver cirrhosis is associated with distinct changes in circulating EVs. In ACLF, EVs are less differentiated and induce mitochondrial dysfunction, decreased T cell viability and changes in the composition of T cell populations.

## Introduction

Liver cirrhosis is a process of tissue scarring by chronic damage leading to loss of liver function. Whereas patients with compensated liver cirrhosis are largely asymptomatic, patients with decompensated liver cirrhosis suffer from complications such as ascites or hepatic encephalopathy (1). Survival rates decrease strongly after transition to decompensated cirrhosis, which is partially explained by an increased risk to develop infections or acute-on-chronic liver failure (ACLF) (2, 3). ACLF is characterized by defined organ failures, excessive systemic inflammation, as well as by a high short-term mortality (4–6).

Liver cirrhosis and in particular ACLF is characterized by a dysfunctional immune response, systemic inflammation and impaired adaptive immunity (7, 8). For example, frequencies of naïve and effector T cells are decreased whereas central memory and effector memory CD4^+^ and CD8^+^ T cells are increased in patients with advanced cirrhosis and ACLF (9). Among other mechanisms, mitochondrial dysfunction may contribute to the immune-pathogenesis and organ failures of ACLF (10).

Extracellular vesicles (EVs) are nano-sized cell derived vesicles with a bi-lipid membrane (11, 12). Regarding their size and biogenesis pathway, EVs are divided into three subgroups: Exosomes (40–150 nm) are released by fusion of multivesicular bodies (MVBs) with the plasma membrane (12, 13); microvesicles (100-1,000 nm) are formed by outward budding and fission of the cellular membrane (14); whereas apoptotic bodies (1,000-5,000 nm) are originated from apoptotic cells by plasma membrane blebs (15). EVs function as contact-independent communicators between cells (12). In the last years, EVs secreted by different cell types like granulocytes, macrophages or apoptotic cells have been found to modulate immune responses (16–18). Yet, the role of EVs in the pathogenesis of ACLF is incompletely understood.

In the present study, we aimed to characterize phenotypes and immune-modulatory functions of the EV compartment in patients with liver cirrhosis through the entire spectrum of the disease from compensated liver cirrhosis to ACLF.

## Material and methods

### Patients

A total number of 84 adult patients diagnosed with liver cirrhosis with or without ACLF were recruited between 2018 and 2021 at the Department of Gastroenterology, University Hospital Essen. Written informed consent was obtained from all participants and human biological samples and related data were provided by the Westdeutsche Biobank Essen (WBE, University Hospital Essen, Essen, Germany; approval WBE-071). Acute decompensation or ACLF were classified according to the criteria of the CLIF-EASL consortium. Pregnant and breast-feeding patients, as well as patients with hepatocellular carcinoma beyond Milan criteria or patients tested positive for human Immunodeficiency virus were excluded from the study. Blood of healthy donors was provided by the blood donation center at the University Hospital Essen. Biographic details of healthy donors were anonymized, so no matching of age and gender could have been performed.

### Isolation of peripheral blood mononuclear cells

For isolation of peripheral blood mononuclear cells (PBMCs), blood of healthy donors, and of patients with liver cirrhosis with or without ACLF was collected. After centrifugation at 3,000 x g for 15 min at 4 °C, plasma was collected and stored at -80 °C. Upon dilution with phosphate buffered saline (PBS; Gibco, Thermo Fisher Scientific, Waltham, USA) the blood cell compartment was layered on top of PANcoll solution (Pan Biotech, Aidenbach, Germany). After centrifugation at 600 x g for 20 min at 4 °C without brake, PBMCs located in the interphase were carefully aspirated and washed twice with PBS at 300 x g for 10 min at 4 °C. Afterwards, PBMC were stored in fetal bovine serum (Sigma, Taufkirchen, Germany) containing 10% DMSO (Sigma, Taufkirchen, Germany) at -80 °C.

### Isolation and characterization of extracellular vesicles

EVs were isolated from plasma of healthy donors, and of patients with liver cirrhosis with or without ACLF. Blood for EV isolation was drawn at baseline of hospitalization. EVs were isolated from EDTA K3 blood taken during routine blood draw in the early morning hours with butterfly system. Tubes were inverted 8–10 times and stored vertically until centrifugation at 3000 xg for 15 min at 4 °C. Plasma was stored at -80 °C until usage. EVs were isolated with Exoquick™ (System Biosciences, Palo Alto, USA) according to the manufacturer’s instructions. Pelleted EVs were resuspended in 0.9% sodium chloride (B. Braun, Melsungen, Germany) supplemented with 1% Penicillin/Streptavidin (Gibco, Thermo Fisher Scientific, Waltham, MA USA). EV fractions were characterized according to the MISEV guidelines (19) to verify the identity of vesicles including (i) determination of particle size, which should be around 150nm, and concentration by nanoparticle tracking analysis with a ZetaView Laser Scattering Video Microscope (ParticleMetrix GmbH, Meerbusch, Germany), (ii) negative staining by Phosphotungstic acid (w/v Carl Roth, Karlsruhe, Germany) in 1.5% aqueous solution of adherent particles on a formvar coated copper grid, (PLANO GmbH, Wetzlar, Germany) followed by transmission electron microscopy (TEM) using JEM 1400Plus (JOEL, Freising, Germany) to visualize the outer of vesicles, (iii) quantification of protein concentration of particles via Pierce™ BCA Protein Assay Kit (Thermo Fisher Scientific, Waltham, MA USA), and (iv) detection of exosome-specific markers with the ExoAb Antibody Kit (System Biosciences, Palo Alto, USA). Furthermore, EVs were characterized via flow cytometry to investigate their cellular origin. In detail, EVs were analyzed via flow cytometry (CytoFLEX S, Beckman Coulter, Brea, CA) for immune cell markers using the MACSPlex Exosome Kit (Miltenyi Biotec, Bergisch Gladbach, Germany; negative control: EV storage buffer) and for markers reflecting liver cells (according to Spittler (20); control: fluorescent Megamix-Plus SSC and Megamix-Plus FSC beads (BioCytex a Stago group company, Marseille, France)) using antibodies against CD11b (BV510 clone ICRF44), CD31 (PE-Cy7 clone WM59), CD68 (APC-Cy7 clone Y1/82A), CD108 (PE clone MEM-150), CD235a (FITC clone ICRF44), all provided by Biolegend (San Diego, CA), and ASGPR (BV650 clone 8D7), CD248 (BV605 clone B1/35), provided by BD Biosciences (San Jose, CA). Finally, the content of small RNAs of EVs was characterized by Illumina NextSeq500 deep sequencing by GenXPro (Frankfurt am Main, Germany) using the TrueQuant method to eliminate PCR artefacts. False discovery rate (FDR) was calculated with Benjamini-Hochberg method and small RNA hits with FDR > 5% were excluded.

### Functional assays to assess the impact of EVs on PBMCs

For functional analysis, exosome-depleted FBS was generated by ultra-centrifugation at 100,000 x g for 130 min at 4 °C (rotor Ti45; Beckman Coulter, Krefeld, Germany). Exosome-free FBS was sterile filtrated and frozen at -30 °C until usage. PBMCs were seeded in RPMI media supplemented with exosome-depleted FBS in 6 well plates. After 2h incubation at 37 °C and 5% CO_2_, 10 µg EVs of healthy donors or patients per 1x10^6^ PBMCs were added. EV-primed PBMCs were harvested after 24h and stained with monoclonal fluorochrome-bound antibodies targeting CD3 (FITC clone UCHT1; AF700 clone UCHT1), CD4 (AF700 clone RPA-T4; BV605 clone OKT4), CD8 (APC/Fire750 clone RPA-T8), CD45RA (PE/Dazzle594 clone HI100), CD183 (PerCP Cy5.5 clone G025H7), or CD196 (PE clone G034E3), CD197 (PE/Cy7 clone G043H7). Cells were additionally analyzed for their Annexin V signal (PE) and viability was assessed with Zombie Aqua staining. All antibodies were provided by BioLegend/San Diego, CA). Mitochondrial function was measured using 50 nM MitoSpy NIRDilC1 (Biolegend, San Diego, CA) and 25 nM MitoTracker Orange CMH2TMROS (ThermoFisher, Waltham, MA USA). Samples were measured using a CytoFlexS cytometer (Beckman Coulter, Brea, CA) with corresponding CytExpert software (Beckman Coulter, Brea, CA). Analysis of data was done using FlowJo v10.7.1.

### Viability assay of CD3^+^ T cells

Healthy donor PBMCs were used for positive T cells isolation using CD3^+^ magnetic beads (Miletnyi Biotec, Bergisch Gladbach, Germany). 20,000 CD3^+^ T cells were seeded in 96 well plates and primed with EVs for 2h at 37 °C and 5% CO_2_. Cells were primed with EVs with or without additional stimulation with 100 µg/ml heparin (Sigma Taufkirchen, Germany) and incubated for 24h at 37 °C and 5% CO_2_ before adding the WST-1 reagent (Sigma, Taufkirchen, Germany). After 4h, the signal was detected at 400 nm wavelength (660 nm reference wavelength) using FLUOstar^®^ Omega (BMG Labtech, Ortenberg, Germany) with the Omega Reader Control software and MARS Data Analysis Software.

### Statistical analysis

Statistical analysis was performed using GraphPad Prism v9.0.2 software (GraphPad Software, San Diego, CA, USA). All metric parameters were given as mean ± SEM. After testing for Gaussian distribution, two groups were tested by T-test or Wilcoxon-Mann-Whitney-U-Test, as appropriate, while comparison of more groups was done by One-way ANOVA or Kruskal-Wallis-Test, as appropriate.

## Results

### Characteristics of included patients

Eighty-four patients with liver cirrhosis were included in this study, of whom 21, 48 and 15 had compensated liver cirrhosis, acute decompensation, or ACLF, respectively (**Table 1**). Etiology of liver cirrhosis was viral hepatitis in 10 patients, (11.90%), non-alcoholic fatty liver disease in 9 patients (10.71%), alcoholic liver disease in 42 patients (50.00%), and cholestatic liver disease in 11 patients (11.90%). In addition, EVs from 20 healthy controls were analyzed.

**Table 1:** Patient characteristics.

	Compensated cirrhosis (N = 21)	AD (N = 48)	ACLF (N = 15)	P-value (comp. vs. AD)	P-value (comp. vs. ACLF)	P-value (AD vs. ACLF)
General characteristics						
Age [years], mean (SD)	56.6 (13.30)	58.9 (9.50)	62.9 (10.15)	0.4	0.2	0.4
Male gender, N (%)	11 (52.4)	28 (58.3)	11 (73.3); 4 (26.7)	0.6	0.2	0.3
Child Pugh Score, mean (SD)	5.24 (0.44)	8.23 (1.17)	9.60 (1.60)	<0.0001	<0.0001	0.09
CLIF OF score, mean (SD)	6.10 (0.30)	6.85 (1.09)	9.27 (1.44)	0.02	<0.0001	<0.0001
MELD score, mean (SD)	8.12 (3.00)	14.4 (5.20)	21.2 (7.88)	0.0001	<0.0001	0.02
Etiology of liver cirrhosis						
Viral, N (%)	3 (14.3)	3 (6.25)	4 (26.7)	0.4	0.4	0.05
NASH, N (%)	2 (9.52)	4 (8.33)	3 (20.00)	>0.99	0.6	0.3
Alcoholic, N (%)	5 (23.8)	29 (60.4)	8 (53.3)	0.008	0.09	0.8
Cholestatic, N (%)	5 (23.8)	5 (10.4)	0 (0.00)	0.2	0.06	0.3
Others, N (%)	6 (28.6)	7 (14.6)	0 (0.00)	0.2	0.03	0.2
Clinical biochemistry						
Leukocytes [per nL], mean (SD)	5.25 (1.76)	6.96 (2.96)	5.27 (2.10)	0.03	0.9	0.07
Hemoglobin [g/dL], mean (SD)	11.91 (2.31)	9.85 (1.85)	8.50 (1.84)	0.0004	<0.0001	0.06
Platelets [per nL], mean (SD)	163.5 (70.2)	148.3 (89.0)	77.9 (30.1)	0.6	0.0003	0.003
CRP [mg/dL], mean (SD)	1.04 (1.10)	2.66 (2.08)	3.12 (2.41)	0.0007	0.001	>0.99
Sodium [mmol/l], mean (SD)	138.1 (3.32)	135.8 (4.43)	132.4 (5.38)	0.1	0.001	0.07
Creatinine [mg/dl], mean (SD)	1.00 (0.21)	1.10 (0.34)	2.11 (0.98)	0.7	<0.0001	<0.0001
Bilirubin [mg/dl], mean (SD)	1.04 (0.62)	3.54 (3.71)	6.06 (8.40)	0.0002	0.002	>0.99
AST [U/l], mean (SD)	39.9 (25.6)	64.0 (69.7)	51.8 (38.2)	0.05	0.8	>0.99
ALT [U/l], mean (SD)	47.3 (53.8)	35.96 (26.3)	37.3 (26.1)	>0.99	>0.99	>0.99
GGT [U/l], mean (SD)	119.6 (81.6)	165.2 (203.5)	114.3 (99.6)	>0.99	>0.99	0.8
AP [U/l], mean (SD)	134.8 (92.9)	180.7 (132.1)	141.2 (58.3)	0.1	0.8	>0.99
INR, mean (SD)	1.11 (0.14)	1.35 (0.33)	1.44 (0.43)	0.0004	0.001	>0.99
Albumin [g/dl], mean (SD)	4.39 (0.52)	3.26 (0.64)	2.90 (0.45)	<0.0001	<0.0001	0.2
IL-6 [pg/ml], mean (SD)	3.96 (10.5)	47.98 (76.8)	44.8 (37.5)	0.0005	0.0002	0.5
ACLF grade						
Grade 1, N (%)	–	–	8 (53.3)	–	–	–
Grade 2, N (%)	–	–	7 (46.7)	–	–	–
Grade 3, N (%)	–	–	0 (0)	–	–	–
Complications of liver cirrhosis						
Hepatic encephalopathy						
Grade 0, N (%)	21 (100.00)	41 (85.42)	7 (31.82)	0.09	<0.0001	<0.0001
Grade 1, N (%)	0 (0.00)	4 (8.33)	4 (26.67)	0.3	0.02	0.08
Grade 2, N (%)	0 (0.00)	2 (4.17)	1 (6.67)	>0.99	0.4	0.6
Grade 3, N (%)	0 (0.00)	1 (2.08)	3 (20.00)	>0.99	0.06	0.03
Gastrointestinal bleeding						
N (%)	0 (0.00)	5 (10.42)	3 (20.00)	0.3	0.06	0.4
Infections						
N (%)	1 (4.76)	13 (27.08)	11 (73.33)	0.05	<0.0001	0.002
Ascites						
No ascites, N (%)	19 (90.48)	15 (31.25)	0 0.00)	<0.0001	<0.0001	0.01
Moderate, N (%)	2 (9.52)*	29 (60.42)	9 (60.00)	<0.0001	0.002	>0.99
Massive, N (%)	0 (0.00)	14 (29.17)	6 (40.00)	0.004	0.002	0.5
Esophageal varices						
Grade 0, N (%)	10 (47.62)	15 (31.25)	4 (26.67)	0.3	0.3	>0.99
Grade 1, N (%)	4 (19.05)	21 (43.75)	3 (20.00)	0.06	>0.99	0.1
Grade 2, N (%)	4 (19.05)	7 (14.58)	7 (46.67)	0.7	0.1	0.02
Grade 3, N (%)	3 (14.29)	5 (10.42)	1 (6.67)	0.6	0.6	>0.99
Outcome						
Mortality within 481 days, N (%)	02 (9.52)	9 (18.75)	9 (60.00)	0.3	0.001	0.0004

*Patients had ascites grade 1 and a child Pugh score of 6 points.

### Morphology and composition of EV-fraction in patients with liver cirrhosis

**Figure 1:**
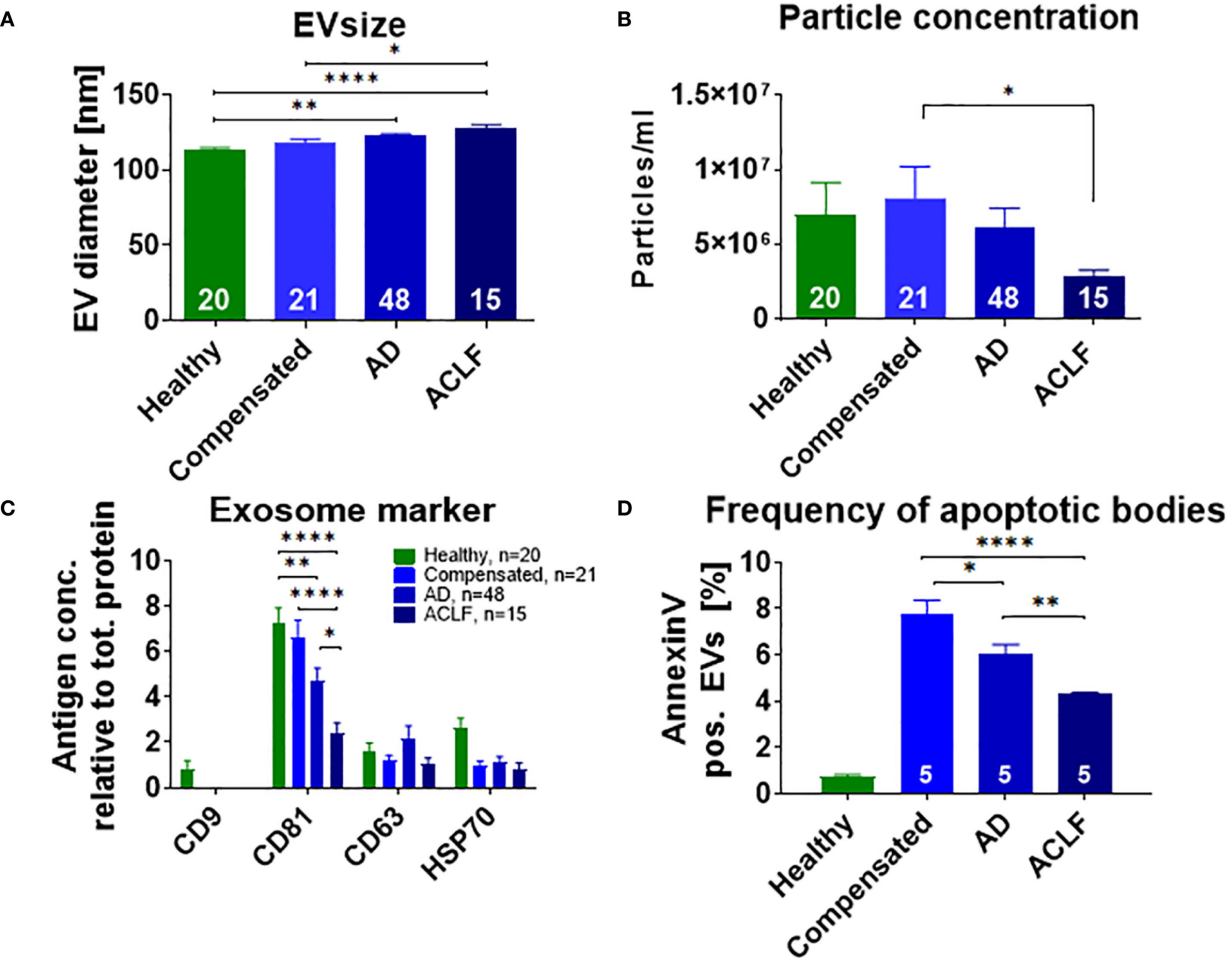
Distinct size and phenotype of EVs in patients with liver cirrhosis. Mean diameter **(A)** and concentration **(B)** of EVs of healthy donors or of patients with liver cirrhosis were assessed by nanoparticle tracking analysis. **(C)** Expression of exosome-specific proteins was quantified by Western blot analysis using antibodies against the indicated antigens. **(D)** For quantification of apoptotic bodies, Annexin V on EVs of healthy controls and patients with liver cirrhosis was measured with flow cytometry. Comparisons with EVs from healthy controls not indicated in the graph (P ≤ 0.0001). Data are presented as mean with SEM. Statistical significance was determined by One-way ANOVA or Kruskal-Wallis test, as appropriate. *P ≤ 0.05, **P ≤ 0.01, ***P ≤ 0.001, ****P≤ 0.0001.

After isolation, purity of EVs was confirmed by transmission electron microscopy and Western Blot analysis for GM130, a marker for cellular debris (**Supplementary Figure S1**). Particle size and concentration of EVs were determined by Nanoparticle tracking analysis. EVs of patients with liver cirrhosis were larger than those of healthy donors and EV size increased with disease progression (healthy control 113 nM, compensated liver cirrhosis 118 nM, acute decompensation 122 nM, ACLF 128 nM, p<0.0001; **Figure 1A**). In contrast, particle concentration of EVs was not significantly higher in patients with compensated liver cirrhosis and acute decompensation compared to healthy donors (6.12x10^6^ vs. 5.30x10^6^ vs. 5.57x10^6^ particles/ml, p>0.9999), whereas it decreased in ACLF (3.44x10^6^ particles/ml, p=0.05; **Figure 1B**).

Next, exosome-specific markers such as CD9, CD63, CD81 and HSP70 were quantified to further characterize EV fractions. In particular, a progressive decline in the frequency of CD81 positive EVs was observed from healthy controls vs. compensated liver cirrhosis vs. acute decompensation vs. ACLF (**Figure 1C**). CD9 was only detectable on EVs of healthy donors, while non-significant trends of lower frequencies of HSP70 in patients with cirrhosis versus healthy controls were observed. A distinct pattern was found for the frequency of EVs positive for Annexin V, a surrogate marker for apoptotic bodies. Patients with liver cirrhosis had higher frequencies of Annexin V positive EVs, which, however, declined from compensated cirrhosis to acute decompensation and ultimately to ACLF (**Figure 1D**).

Since the etiology of liver cirrhosis in our cohort was heterogenous, we next assessed the impact of bacterial infections and etiology on particle size and expression of surface antigens. (**Supplementary Figure S2** and **S3**). Overall, a moderate impact of bacterial infection and etiology on these parameters was observed.

### Cellular source and RNA content of EVs

**Figure 2:**
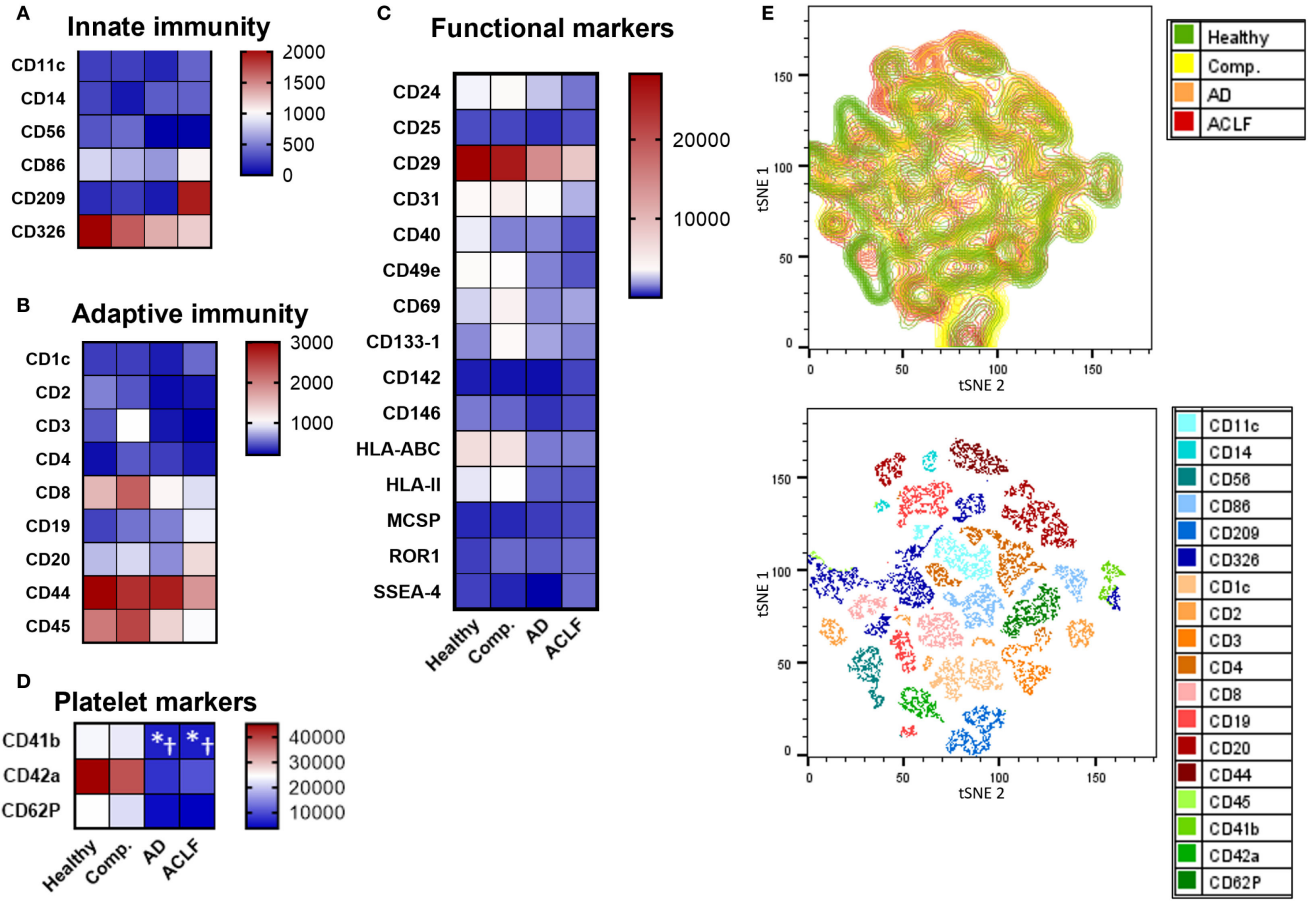
Most immune cell markers are not enriched in EVs of patients with liver cirrhosis. **(A-D)** To determine the cellular origin of EVs, EVs from patients or controls were stained with antibodies against specific cell surface markers and quantified by flow cytometry (N = 5) using a MACSPlex Kit. **(E)** tSNE visualization of MACSPlex measurement by FlowJo and tSNE plugin. UMAP analysis was performed on 18 surface markers for 24 EV samples from healthy donors or patients with cirrhosis with or without ACLF (N = 6 per group). UMAP plot from patient groups shows the relative abundance and UMAP plot for all included markers are displayed. Statistical significance was determined by One-way ANOVA or Kruskal-Wallis test, as appropriate. *P ≤ 0.05 significance to healthy control, †P ≤ 0.05 significance to compensated sample.

To determine the cellular source of EVs according to the stage of liver disease, markers specific for important immune cell populations, platelets, and liver cells were determined on isolated EVs by flow cytometry. The expression of immune cell markers on EVs was not significantly different across the stages of liver cirrhosis, but numerically most immune cell markers declined while some increased (e.g. CD20) in patients with liver cirrhosis compared to healthy controls and with progressive severity of liver disease (**Figures 2A-C**). The expression of platelet markers also decreased with increasing severity of liver cirrhosis (**Figure 2D**).

**Figure 3:**
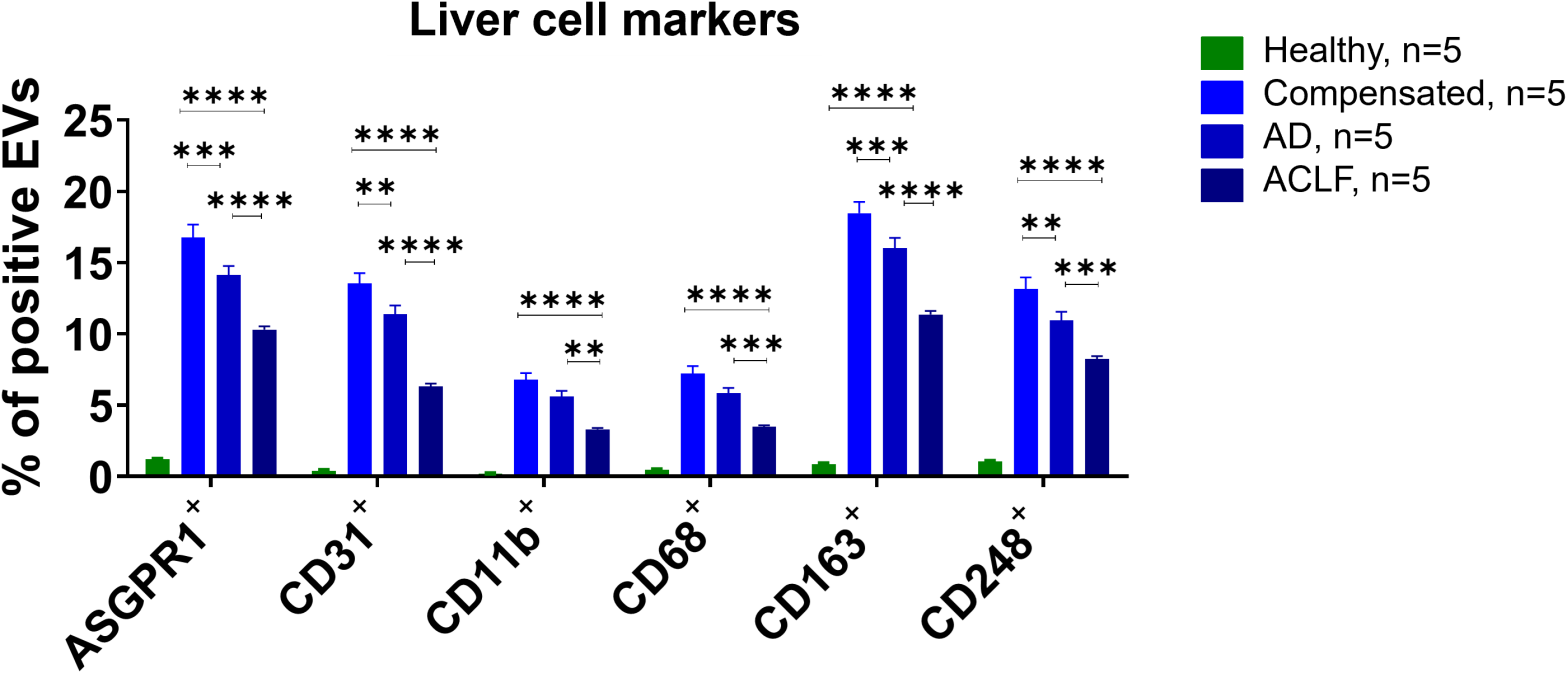
Liver cell markers are enriched in EVs of patients with liver cirrhosis. To determine the cellular origin of EVs, EVs from patients or controls were stained with antibodies against specific cell surface markers and quantified by flow cytometry (N = 5). All data are presented as mean with SEM. Statistical significance was determined by two-way ANOVA. **P ≤ 0.01, ***P ≤ 0.001, ****P ≤ 0.0001; comparisons with EVs from healthy controls not indicated in the graph (P ≤ 0.0001 for all markers).

A contrasting pattern of markers suggesting an origin from hepatocytes (ASGPR1), liver sinusoidal endothelial cells (CD31), Kupffer cells (CD11b, CD68, CD163), and hepatic stellate cells (CD248) was observed. Profoundly higher frequencies of EVs with a liver cell signature were observed in patients with liver cirrhosis compared to healthy controls, though their relative abundance declined with disease severity (**Figure 3**). Again, moderate differences of frequencies of liver EVs were observed according to the presence of infections and etiology of liver cirrhosis (**Supplementary Figures S2** and **S3**).

**Figure 4:**
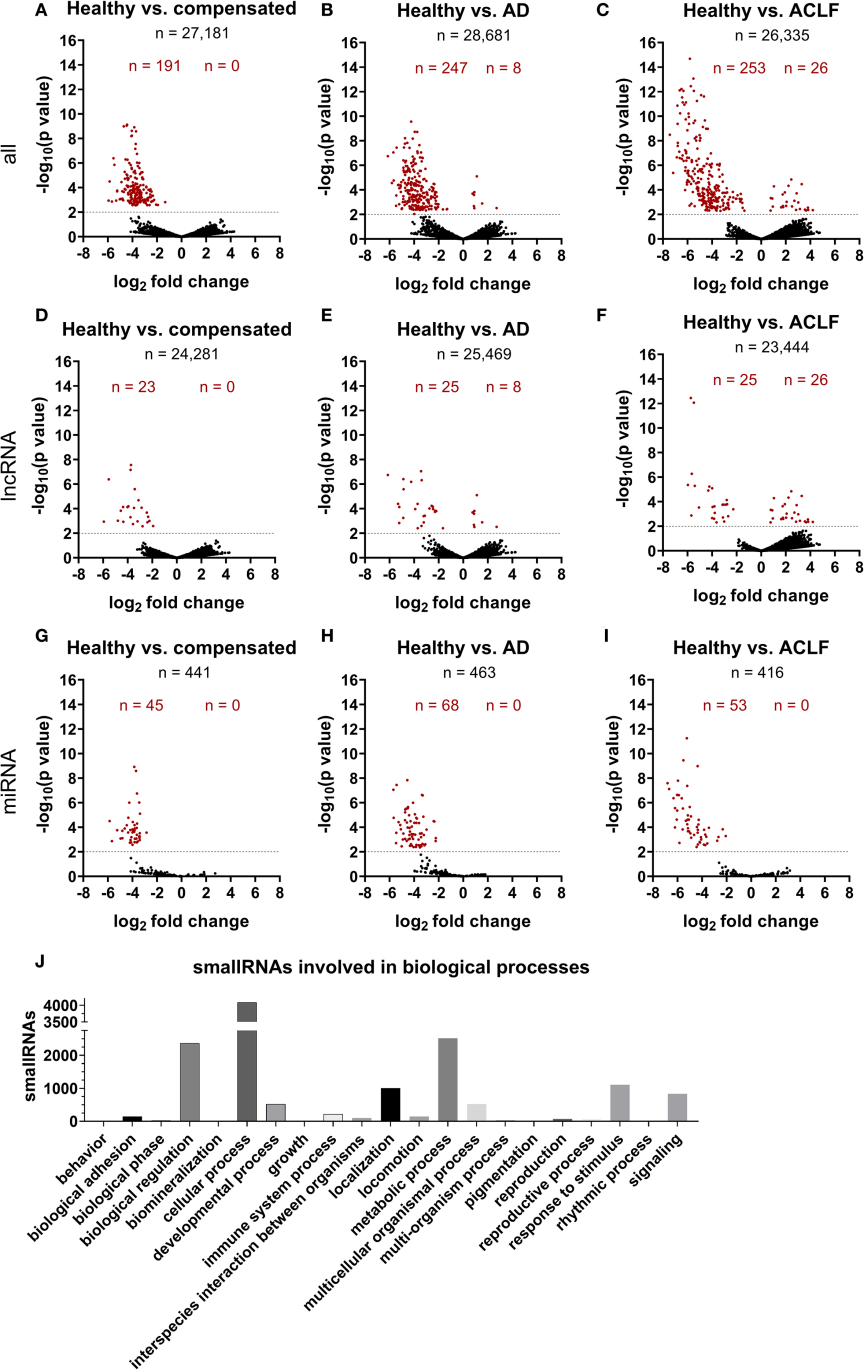
SmallRNA cargo of EVs is distinct in liver cirrhosis and ACLF. Illumina NextSeq500 deep sequencing was used by GenXPro (Frankfurt am Main, Germany) to characterize smallRNA cargo of EVs **(A-J)**. Log_2_fold change was determined using the program DE-Seq2 **(B-J)**. Threshold of –log10(p value) was set on 2, corresponding a P-value of 0.01. Red-marked points are significantly different expressed smallRNAs. Negative log2 fold changes refer to higher expression of regarded smallRNA in group 2 compared to group 1. K) Pathway analysis of all detected smallRNAs was performed using PANTHER tool (33, 34).

Additionally, small RNA sequencing was performed to characterize RNA signatures of EVs (**Figure 4**, **Supplementary Tables S1** and **S2**). In total, the number of detectable small RNAs species decreased notably with disease progression (**Supplementary Table S1**). This applied in particular for long-coding RNAs, and micro RNAs (**Supplementary Table S1**). A number of 191, 255 and 279 small RNAs were significantly different in concentration of EVs of patients with compensated cirrhosis, acute decompensation or ACLF, compared to healthy controls (**Figure 4**). Pathway analysis revealed a large number of RNA species involved in cellular metabolism (**Figure 4**). Furthermore, significant changes in the amount of small RNAs, which were previously described in mitochondrial function, were detected (**Supplementary Table S3**).

### EVs of patients with liver cirrhosis induce relative shifts in T cell subpopulations

**Figure 5:**
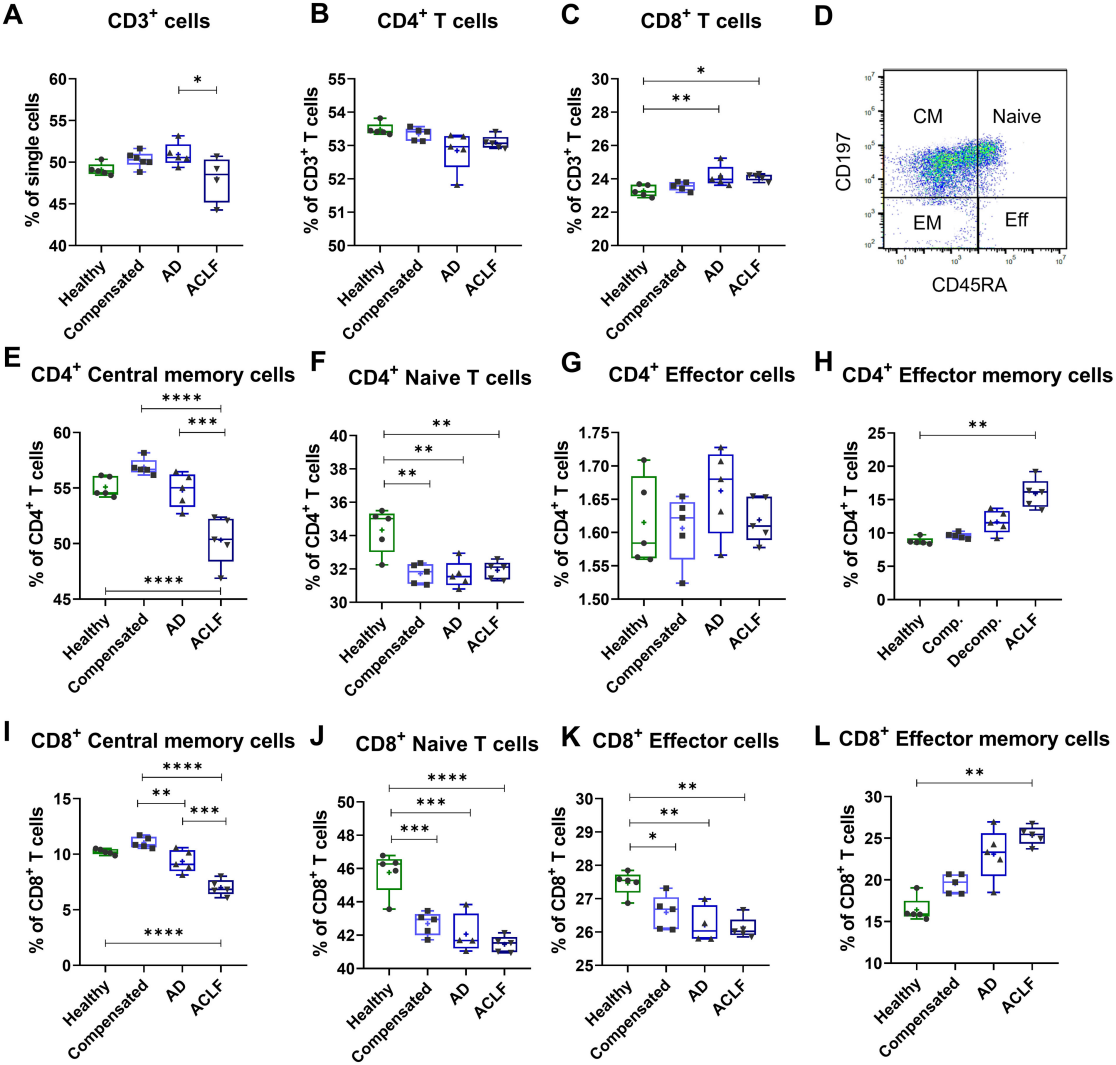
EVs of patients with liver cirrhosis induce shifts in T cell populations. PBMCs of healthy donors (N = 6) were primed with EVs of different healthy controls or patients for 24 h. T cell populations were quantified by flow cytometric analysis for total CD3 T cells **(A)**, total CD4^+^ T cells **(B)**, total CD8^+^ T cells **(C)**, as well as for CD4^+^ T cell subpopulations **(E-H)** and CD8^+^ T cell subpopulations **(I-L)**. **(D)** Representative FACS plot showing a CD45RA/CD197 T-cell staining from one exemplary sample. Data are presented as median with the 10th and 90th percentile. Statistical significance was determined by One-way ANOVA or Kruskal-Wallis test, depending on normality test. *P ≤ 0.05, **P ≤ 0.01, ***P ≤ 0.001, ****P ≤ 0.0001.

In functional assays, PBMCs from healthy donors were stimulated with 20 µg EVs derived from patients of different liver cirrhosis stages for 24h and proportions of different T cell subpopulations were quantified *via* flow cytometry. EVs of patients with liver cirrhosis induced relative changes in the composition of T cell populations. In detail, the amount of all living CD3^+^ T cells decreased after incubation with EVs of patients with ACLF (**Figure 5A**). More specifically, the frequency of total CD4^+^ T cells did not change (**Figure 5B**) whereas the frequency of CD8^+^ T cells significantly increased after stimulation with EVs from patients with liver cirrhosis (**Figure 5C**). Furthermore, EVs led to changes in the composition of subpopulations by inducing a loss of central memory and naïve CD4^+^ and CD8^+^ T cells, while also causing an increase in the frequency of effector memory T cells (**Figures 5E-L**).

### EVs of patients with ACLF induce cell stress and mitochondrial dysfunction in T cells

**Figure 6:**
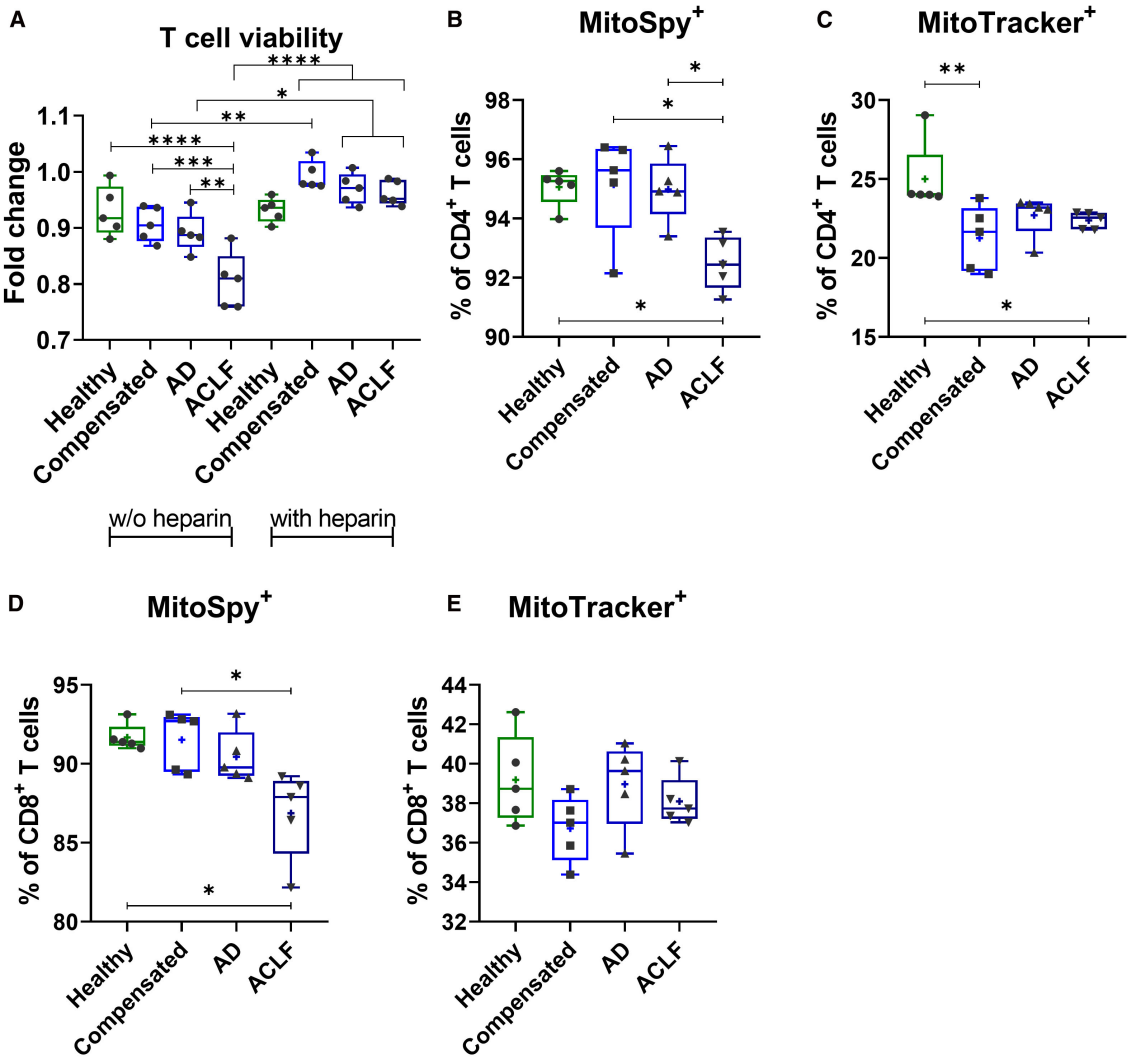
EVs of patients with liver cirrhosis impact T cell viability and induce mitochondrial dysfunction. **(A)** CD3^+^ T cells of healthy donors (N = 6) were isolated and primed with EVs derived from different healthy donors or liver cirrhosis patients without (w/o) or with additional stimulation with heparin for 24h. T cell viability was assessed by WST-1 assay. PBMCs of healthy donors (N = 6) were stimulated with either EVs of different healthy donors or of patients with liver cirrhosis for 24h. Frequencies of MitoSpy-positive cells, reflecting mitochondrial abundance [MitoSpy; **(B, D)**] and MitoTracker-positive cells, indicating mitochondrial functionality [MitoTracker; **(C, E)**] were analyzed via flow cytometry. Data are presented as median with the 10th and 90th percentile. Statistical significance was determined by **(A-F)** One-way ANOVA or Kruskal-Wallis test depending on normality test. *P ≤ 0.05, **P ≤ 0.01, ***P ≤ 0.001.

Incubation of T cells with EVs of patients with liver cirrhosis and – in particular – with ACLF resulted in decreased T cell viability, as assessed by the WST-1 assay (**Figure 6A**). Heparin, previously described to block EV binding and uptake of target cells (21), was used as a control. T cells with simultaneous EV and heparin stimulation show no changes in T cell viability. To understand possible mechanisms, mitochondrial presence and function was determined via staining with MitoSpy and MitoTracker and quantification by flow cytometry. Incubation of PBMCs with EVs from patients with ACLF resulted in reduced numbers of mitochondria in CD4^+^ and CD8^+^ T cells compared to cells stimulated with EVs from healthy donors or with EVs of patients with compensated liver cirrhosis (**Figures 6B, D**). In addition, a trend of reduced mitochondrial function of CD8^+^ T cells (**Figure 6E**) whereas a significant reduction was observed in CD4^+^ T cells after stimulation with EVs from patients with liver cirrhosis, a phenomenon which was again most pronounced in ACLF (**Figure 6C**).

## Discussion

The main findings of our study demonstrate that EVs of patients with liver cirrhosis differ from those of healthy individuals with respect to their cellular source, their phenotype, and RNA cargo. These alterations are mostly pronounced in ACLF. Interestingly, EVs from patients with ACLF induce cell stress and mitochondrial dysfunction in T cells, which may affect T cell viability in advanced liver cirrhosis and may therefore contribute to shifts in T cell subpopulations.

According to our study, EVs of patients with compensated liver cirrhosis secrete slightly higher total amounts of EVs than healthy individuals, whereas in patients with advanced liver disease, in particular with ACLF, the concentration of EVs in blood declines significantly. These findings were somehow unexpected, because in previous studies higher concentrations of EVs in cirrhosis, and in particular in patients with severe alcoholic hepatitis, have been described (22). Of note, a previous study involving patients with ACLF has shown decreasing concentrations of EVs as well, consistent with our study (23). Conflicting results in previous studies may be attributed to the applied isolation method (24). In this regard, a previous recommendation of standardized isolation methods is crucial for the successful use of EVs as biomarkers for specific liver disease (24). Yet, our finding of declining EV-concentrations in patients with ACLF is of interest, because data on EVs in ACLF are scarce. One may speculate that in these very sick patients EVs are unstable due to toxic alterations of lipid membranes, which may result in a shorter half live *in vivo* (or in a decline during the isolation progress). A consistent finding to most previous study is the increasing size of EVs with disease progression (25).

For both diagnostic and functional aspects, phenotype and cargo of EVs are likely more important than their concentration. In this regard, our study shows profound differences in the cellular source, protein- and RNA-cargo of EVs in liver cirrhosis. In particular, the proportion of EVs from various resident liver cells including hepatocytes, endothelial cells or Kupffer cells is strongly increasing in patients already with compensated liver cirrhosis, whereas the proportion of EVs from the majority of circulating immune cells is rather decreasing. A previously published large biomarker study showed an increase in EVs of hepatic origin to be associated with poor prognosis in patients with compensated, alcohol-related liver disease (26). In general, EVs are secreted from cells including hepatocytes in response to various stimuli (25). Hence, in the setting of profound systemic inflammation, an increase of EVs from immune cells in ACLF might have been expected. Yet, previous studies have shown a significant decline and functional exhaustion of many immune cell populations in ACLF, which may explain the decreasing concentration of immune cell-derived EVs in patients with ACLF (9). Notably, the frequency of EVs with liver cell signatures declines also in patients with ACLF compared to compensated cirrhosis or acute decompensation (although it is still much higher than in healthy individuals), as well as the proportion of EVs which are positive for exosome-specific markers such as CD81. At present, we can only speculate about the causes of this phenomenon, which may involve an altered secretion machinery or altered sources of EVs in ACLF, but even technical issues due to unstable membranes cannot fully be excluded.

Of note, the changing repertoire of EVs in liver cirrhosis and in particular in ACLF appears to have pathophysiological consequences. Inflammation and cell death are important determinants in the pathogenesis of ACLF, which have been linked with mitochondrial toxicity due to profound metabolic alterations in this entity (10, 27). EVs may play an important role in regulating these processes. In our study, EVs from patients with liver cirrhosis reduced T cell viability and induced mitochondrial dysfunction – findings which were most pronounced for EVs from patients with ACLF. Mechanistically, mitochondrial toxicity of EVs might be mediated by specific microRNAs or lncRNAs like let7b-5p, which have been recently recognized to regulate mitochondria (28–31), **Supplementary Table S2**. In addition, EVs from patients with cirrhosis induced profound changes in the composition of T cell populations *ex vivo*, which may be functionally linked to their impact on cell viability and mitochondrial function. Of note, the induced changes – namely a decrease of naïve T cells and central memory T cells and an increase of effector memory T cells – resembled to the altered *in vivo* T cell compartment in patients with ACLF (9). Collectively, our data suggest that EVs contribute to key determinants – immune dysfunction, cell death and mitochondrial toxicity – in the pathogenesis of ACLF. This assumption is in line with a recent study in mice showing that EVs in alcoholic hepatitis can cause dysfunction in skeletal muscle which may contribute to the pathogenesis of sarcopenia (32).

Our study has some limitations. The sample size of our study was too small for a detailed analysis of the impact of etiology of liver cirrhosis on EV phenotype and function in ACLF, which may have an impact for example on protein and RNA cargo of EVs (25). Furthermore, the precise mechanism of inducing mitochondrial dysfunction remains to be established in detail in future studies. Finally, EVs from healthy controls used for our study were derived from individuals with anonymized biographic details. Hence, there is a risk of a bias with respect to an insufficient matching for age and gender.

Collectively, our study reveals profound phenotypic and functional alterations of EVs in patients with liver cirrhosis and in particular with ACLF, which may contribute to the pathogenesis of ACLF by inducing mitochondrial toxicity and immune dysfunction.

## Data Availability

The original contributions presented in the study are included in the article/**Supplementary Material**. Further inquiries can be directed to the corresponding author.

## References

[1] D’Amico G Garcia-Tsao G Pagliaro L. Natural history and prognostic indicators of survival in cirrhosis: A systematic review of 118 studies. J Hepatol. (2006) 44:217–31. doi: 10.1016/j.jhep.2005.10.013, PMID: 16298014

[2] Gustot T Durand F Lebrec D Vincent JL Moreau R. Severe sepsis in cirrhosis. Hepatology. (2009) 50:2022–33. doi: 10.1002/hep.23264, PMID: 19885876

[3] Trebicka J Fernandez J Papp M Caraceni P Laleman W Gambino C. The PREDICT study uncovers three clinical courses of acutely decompensated cirrhosis that have distinct pathophysiology. J Hepatol. (2020) 73:842–54. doi: 10.1016/j.jhep.2020.06.013, PMID: 32673741

[4] Moreau R Jalan R Gines P Pavesi M Angeli P Cordoba J. Acute-on-chronic liver failure is a distinct syndrome that develops in patients with acute decompensation of cirrhosis. Gastroenterology. (2013) 144:1426–1437.e1429. doi: 10.1053/j.gastro.2013.02.042, PMID: 23474284

[5] Arroyo V Moreau R Kamath PS Jalan R Ginès P Nevens F. Acute-on-chronic liver failure in cirrhosis. Nat Rev Dis Primers. (2016) 2:16041. doi: 10.1038/nrdp.2016.41, PMID: 27277335

[6] Arroyo V Moreau R Jalan R. Acute-on-chronic liver failure. New Engl J Med. (2020) 382:2137–45. doi: 10.1056/NEJMra1914900, PMID: 32459924

[7] Lange CM Moreau R. Immunodysfunction in acute-on-chronic liver failure. Visceral Med. (2018) 34:276–82. doi: 10.1159/000488690, PMID: 30345285 PMC6189545

[8] Clària J Stauber RE Coenraad MJ Moreau R Jalan R Pavesi M. Systemic inflammation in decompensated cirrhosis: Characterization and role in acute-on-chronic liver failure. Hepatology. (2016) 64:1249–64. doi: 10.1002/hep.28740, PMID: 27483394

[9] Rueschenbaum S Ciesek S Queck A Widera M Schwarzkopf K Brüne B. Dysregulated adaptive immunity is an early event in liver cirrhosis preceding acute-on-chronic liver failure. Front Immunol. (2021) 11. doi: 10.3389/fimmu.2020.534731, PMID: 33574809 PMC7870861

[10] Moreau R Clària J Aguilar F Fenaille F Lozano JJ Junot C. Blood metabolomics uncovers inflammation-associated mitochondrial dysfunction as a potential mechanism underlying ACLF. J Hepatol. (2020) 72:688–701. doi: 10.1016/j.jhep.2019.11.009, PMID: 31778751

[11] Raposo G Stoorvogel W. Extracellular vesicles: exosomes, microvesicles, and friends. J Cell Biol. (2013) 200:373–83. doi: 10.1083/jcb.201211138, PMID: 23420871 PMC3575529

[12] Yáñez-Mó M Siljander PR Andreu Z Zavec AB Borràs FE Buzas EI. Biological properties of extracellular vesicles and their physiological functions. J Extracell Vesicles. (2015) 4:27066. doi: 10.3402/jev.v4.27066, PMID: 25979354 PMC4433489

[13] Maji S Matsuda A Yan IK Parasramka M Patel T. Extracellular vesicles in liver diseases. Am J Physiol Gastrointest liver Physiol. (2017) 312:G194–200. doi: 10.1152/ajpgi.00216.2016, PMID: 28039157 PMC5401990

[14] Muralidharan-Chari V Clancy JW Sedgwick A D’Souza-Schorey C. Microvesicles: mediators of extracellular communication during cancer progression. J Cell Sci. (2010) 123:1603–11. doi: 10.1242/jcs.064386, PMID: 20445011 PMC2864708

[15] Chen Y Li G Liu M-L. Microvesicles as emerging biomarkers and therapeutic targets in cardiometabolic diseases. Genomics Proteomics Bioinf. (2018) 16:50–62. doi: 10.1016/j.gpb.2017.03.006, PMID: 29462670 PMC6000161

[16] Cai C Koch B Morikawa K Suda G Sakamoto N Rueschenbaum S. Macrophage-derived extracellular vesicles induce long-lasting immunity against hepatitis C virus which is blunted by polyunsaturated fatty acids. Front Immunol. (2018) 9:723. doi: 10.3389/fimmu.2018.00723, PMID: 29706960 PMC5906748

[17] Saha B Momen-Heravi F Furi I Kodys K Catalano D Gangopadhyay A. Extracellular vesicles from mice with alcoholic liver disease carry a distinct protein cargo and induce macrophage activation through heat shock protein 90. Hepatology. (2018) 67:1986–2000. doi: 10.1002/hep.29732, PMID: 29251792 PMC5906190

[18] Alexander M Hu R Runtsch MC Kagele DA Mosbruger TL Tolmachova T. Exosome-delivered microRNAs modulate the inflammatory response to endotoxin. Nat Commun. (2015) 6:7321. doi: 10.1038/ncomms8321, PMID: 26084661 PMC4557301

[19] Théry C Witwer KW Aikawa E Alcaraz MJ Anderson JD Andriantsitohaina R. Minimal information for studies of extracellular vesicles 2018 (MISEV2018): a position statement of the International Society for Extracellular Vesicles and update of the MISEV2014 guidelines. J Extracell Vesicles. (2018) 7:1535750. doi: 10.1080/20013078.2018.1535750, PMID: 30637094 PMC6322352

[20] Spittler A. Set-Up of the CytoFLEX for Extracellular Vesicle Measurement. Cytoflex Application Note. Brea, California: Beckman Coulter (2015).

[21] Atai NA Balaj L van Veen H Breakefield XO Jarzyna PA Van Noorden CJ. Heparin blocks transfer of extracellular vesicles between donor and recipient cells. J Neurooncol. (2013) 115:343–51. doi: 10.1007/s11060-013-1235-y, PMID: 24002181 PMC3856724

[22] Sehrawat TS Arab JP Liu M Amrollahi P Wan M Fan J. Circulating extracellular vesicles carrying sphingolipid cargo for the diagnosis and dynamic risk profiling of alcoholic hepatitis. Hepatology. (2021) 73:571–85. doi: 10.1002/hep.31256, PMID: 32246544 PMC7541595

[23] Sanchez-Rodriguez MB Tellez E Casulleras M Borras FE Arroyo V Claria J. Reduced plasma extracellular vesicle CD5L content in patients with acute-on-chronic liver failure: interplay with specialized pro-resolving lipid mediators. Front Immunol. (2022) 13:842996. doi: 10.3389/fimmu.2022.842996, PMID: 35330909 PMC8940329

[24] Thietart S Rautou PE. Extracellular vesicles as biomarkers in liver diseases: A clinician’s point of view. J Hepatol. (2020) 73:1507–25. doi: 10.1016/j.jhep.2020.07.014, PMID: 32682050

[25] Szabo G Momen-Heravi F. Extracellular vesicles in liver disease and potential as biomarkers and therapeutic targets. Nat Rev Gastroenterol Hepatol. (2017) 14:455–66. doi: 10.1038/nrgastro.2017.71, PMID: 28634412 PMC6380505

[26] Elkrief L Ganne-Carrie N Manceau H Tanguy M Valainathan SR Riescher-Tuczkiewicz A. Hepatocyte-derived biomarkers predict liver-related events at 2 years in Child-Pugh class A alcohol-related cirrhosis. J Hepatol. (2023) 79:910–23. doi: 10.1016/j.jhep.2023.05.025, PMID: 37302582

[27] Hernaez R Solà E Moreau R Ginès P. Acute-on-chronic liver failure: an update. Gut. (2017) 66:541–53. doi: 10.1136/gutjnl-2016-312670, PMID: 28053053 PMC5534763

[28] Dragomir MP Knutsen E Calin GA. SnapShot: unconventional miRNA functions. Cell. (2018) 174:1038–1038.e1031. doi: 10.1016/j.cell.2018.07.040, PMID: 30096304

[29] Li H Dai B Fan J Chen C Nie X Yin Z. The Different Roles of miRNA-92a-2-5p and let-7b-5p in Mitochondrial Translation in db/db Mice. Mol Ther - Nucleic Acids. (2019) 17:424–35. doi: 10.1016/j.omtn.2019.06.013, PMID: 31319246 PMC6637210

[30] Li P Jiao J Gao G Prabhakar BS. Control of mitochondrial activity by miRNAs. J Cell Biochem. (2012) 113:1104–10. doi: 10.1002/jcb.24004, PMID: 22135235 PMC3325319

[31] Cavalcante GC Magalhães L Ribeiro-Dos-Santos Â Vidal AF. Mitochondrial epigenetics: non-coding RNAs as a novel layer of complexity. Int J Mol Sci. (2020) 21:1838. doi: 10.3390/ijms21051838, PMID: 32155913 PMC7084767

[32] Barberi L Porcu C Boccia C Cosentino M Nicoletti C Peruzzi B. Circulating extracellular vesicles in alcoholic liver disease affect skeletal muscle homeostasis and differentiation. J Cachexia Sarcopenia Muscle. (2025) 16:e13675. doi: 10.1002/jcsm.13675, PMID: 39921321 PMC11806195

[33] Mi H Thomas P. PANTHER pathway: an ontology-based pathway database coupled with data analysis tools. Methods Mol Biol (Clifton N.J.). (2009) 563:123–40. doi: 10.1007/978-1-60761-175-2_7, PMID: 19597783 PMC6608593

[34] Mi H Ebert D Muruganujan A Mills C Albou L-P Mushayamaha T. PANTHER version 16: a revised family classification, tree-based classification tool, enhancer regions and extensive API. Nucleic Acids Res. (2021) 49:D394–403. doi: 10.1093/nar/gkaa1106, PMID: 33290554 PMC7778891

