# Supplementary material for: Extracellular vesicles of patients with acute-on-chronic liver failure induce mitochondrial dysfunction in T cells
Source: Front Immunol. 2025 Sep 22;16:1656692. doi: 10.3389/fimmu.2025.1656692 (PMC12497775; doi:10.3389/fimmu.2025.1656692)
Supplement: Supplementary file 1 [file DataSheet1.docx]

**Supplementary Figures**


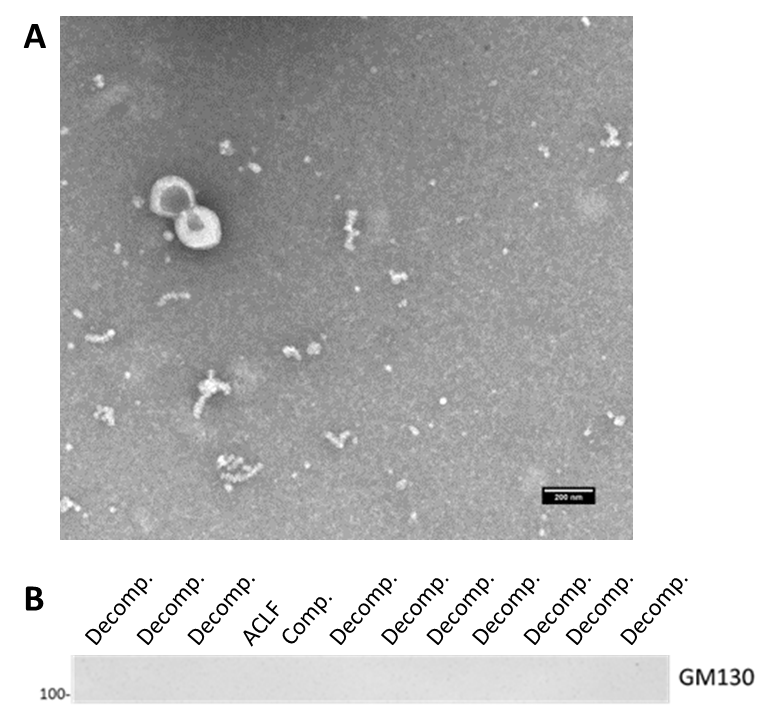


Supplementary Figure 1. Quality controls of EV preparations. A) Transmission electron microscopy was used to visualize EVs. A picture of EVs of a healthy donor is shown exemplarily. B) Western blot analysis of GM130, a marker for cellular debris, was used to verify purity of EVs.


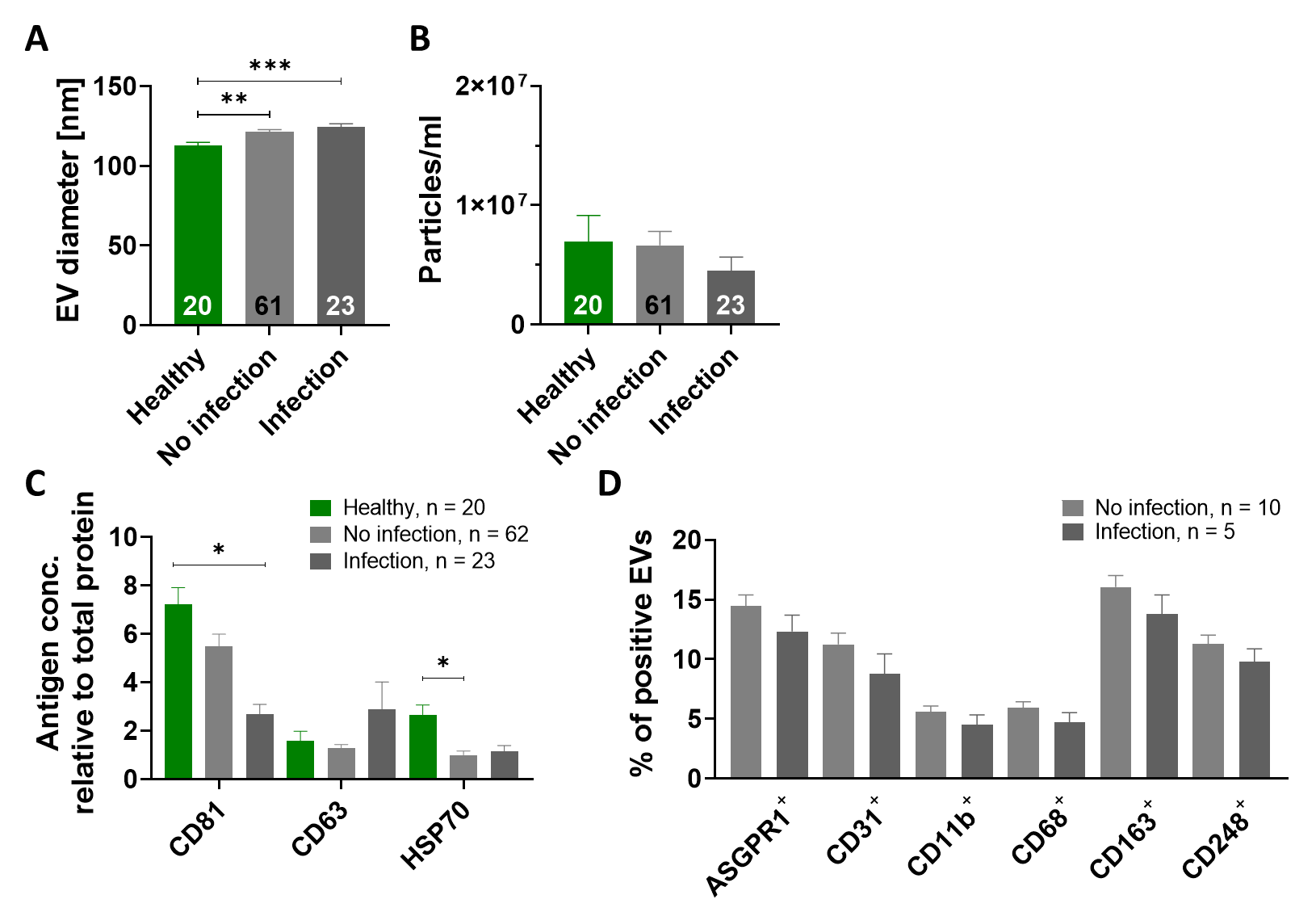


Supplementary Figure 2. Association between bacterial infections and EV phenotype. Nanoparticle tracking analysis was used to determine particle size (A) and concentration (B) of plasma EVs of healthy donors and patients with or without infections. C) EV markers were determined by Western Blot analysis. D) Liver cell surface antigens on EVs were determined by flow cytometry. Data are presented as mean with SEM. Statistical significance was determined by One-way ANOVA or Kruskal-Wallis test, as appropriate. *P ≤ 0.05, **P ≤ 0.01, ***P ≤ 0.001, ****P≤ 0.0001.


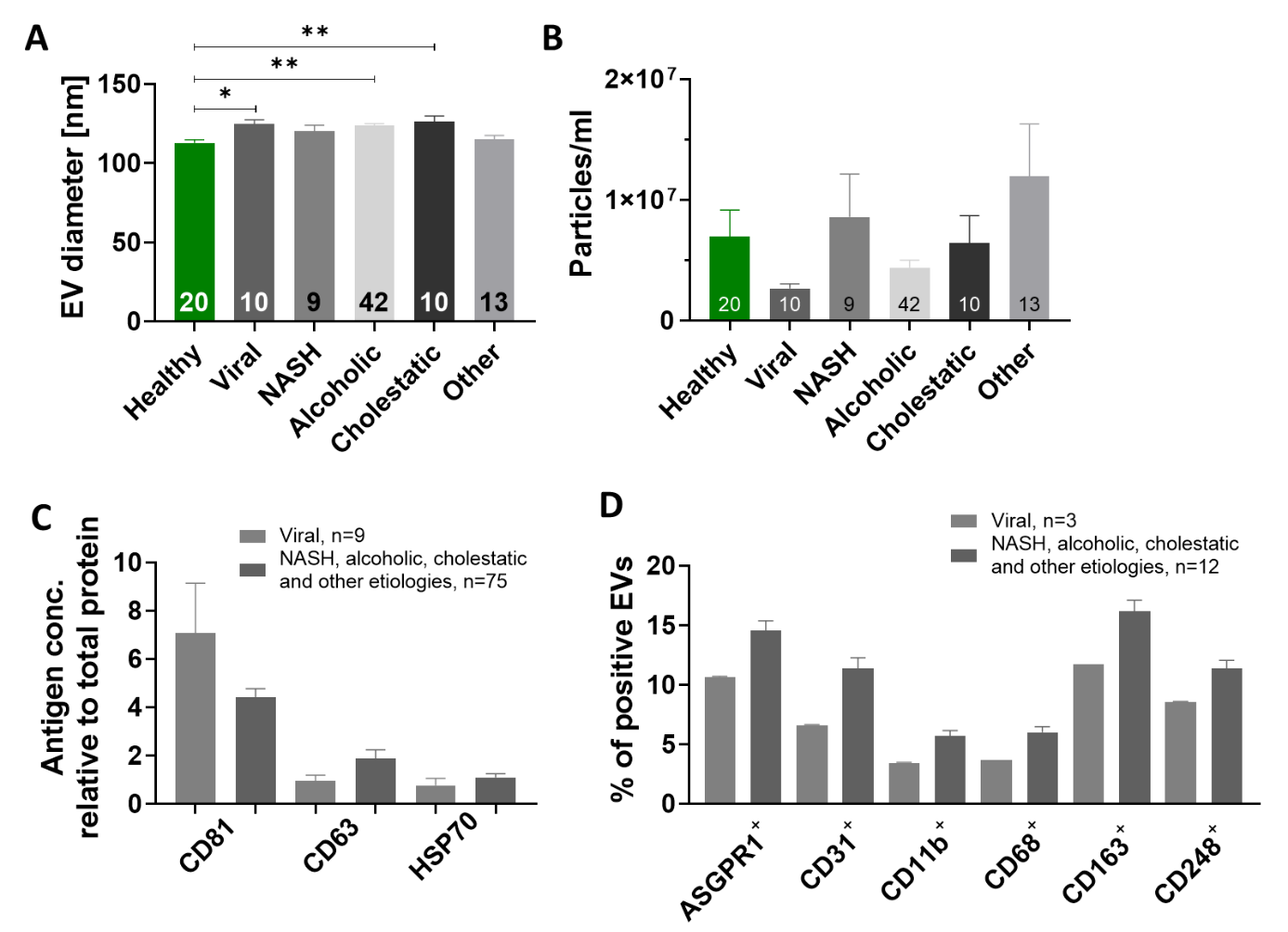


Supplementary Figure 3. Association between etiology of liver cirrhosis and EV phenotype. Nanoparticle tracking analysis was used to determine particle size (A) and concentration (B) of plasma EVs of healthy donors and patients according to the etiology of liver cirrhosis. C) EV markers were determined by Western Blot analysis. D) Liver cell surface antigens on EVs were determined by flow cytometry. Data are presented as mean with SEM. Statistical significance was determined by One-way ANOVA or Kruskal-Wallis test, as appropriate. *P ≤ 0.05, **P ≤ 0.01, ***P ≤ 0.001, ****P≤ 0.0001.

**Supplementary tables**

**Supplementary Table (see separate Excel sheet): RNA cargo of EVs derived from healthy donors or liver cirrhosis patients with or without ACLF.**
